# Differential Impact of SiO_2_ Foliar Application on Lettuce Response to Temperature, Salinity, and Drought Stress

**DOI:** 10.3390/plants14121845

**Published:** 2025-06-16

**Authors:** Ivan Simko, Rebecca Zhao, Hui Peng

**Affiliations:** 1 Sam Farr United States Crop Improvement and Protection Research Center, Agricultural Research Service, U.S. Department of Agriculture, Salinas, CA 93905, USA; rebecca.zhao@usda.gov; 2 Everglades Research and Education Center–Horticultural Sciences Department, University of Florida, Belle Glade, FL 33430, USA; huipeng@ufl.edu

**Keywords:** silicon dioxide, foliar application, abiotic stress, salinity tolerance, lettuce (*Lactuca sativa*), mineral composition, ion homeostasis

## Abstract

Silicon dioxide (SiO_2_) foliar application offers a promising strategy for enhancing lettuce (*Lactuca sativa* L.) resilience under temperature extremes, salinity, and drought stress. This study investigated the effects of SiO_2_ treatment on three lettuce cultivars exposed to varying temperature, salinity, and drought conditions in a controlled growth chamber environment. Silicon treatment (3.66 mM) significantly enhanced plant biomass under suboptimal (15 °C), optimal (20 °C), and salinity stress conditions. Notably, the SiO_2_ effect was most positive under severe salinity stress (100 mM NaCl), where its application increased plant weight together with chlorophyll and anthocyanin content. When increasing SiO_2_ concentrations from 0 to 29.30 mM were tested, optimal results to alleviate severe salinity stress were consistently observed at 3.66 mM, with peak performance in fresh weight, plant diameter, chlorophyll, and anthocyanin content. Higher SiO_2_ concentrations progressively diminished these beneficial effects, with 29.30 mM treatment leading to reduced growth and increased leaf chlorosis. Comprehensive mineral composition analysis revealed complex interactions between silicon treatment and elemental profiles at 100 mM salinity stress. At 3.66 mM SiO_2_, plants accumulated the highest levels of both K (20,406 mg/kg dry weight, DW) and Na (16,185 mg/kg DW) while maintaining the highest K/Na ratio (1.26). This suggests that Si enhances cellular ion compartmentalization rather than exclusion mechanisms, allowing plants to manage higher total ion content better while minimizing cytoplasmic damage. Drought stress conditions unexpectedly revealed negative impacts from 3.66 mM SiO_2_ application, with decreased plant fresh weight at moderate (50% soil water content, SWC) and severe (30% SWC) water limitations, though results were statistically significant only under severe drought stress. The study highlights silicon’s potential as a stress mitigation agent, particularly under salinity stress, while emphasizing the need for concentration-specific and stress-specific approaches. These findings suggest that foliar SiO_2_ application could be a valuable tool for enhancing lettuce crop productivity under both optimal and challenging environmental conditions, with future research warranting field validation and full market maturity assessments.

## 1. Introduction

Lettuce (*Lactuca sativa* L.) is one of the most commercially important leafy vegetables globally, cultivated primarily in moderate climates. It is valued for its versatility in culinary use, predominantly as fresh leaves in salads, though certain types also feature edible stems. In the United States, lettuce production is concentrated in California and Arizona, accounting for over 90% of the national supply [1]. The Salinas Valley in California, often referred to as the “Salad Bowl of America,” is a major hub for lettuce cultivation, contributing significantly to the crop’s total farm gate value, which exceeded $4.6 billion in 2024 [2].

Lettuce cultivars are classified into several horticultural types, including iceberg, romaine, butterhead, and leaf varieties. These types cater to a wide range of consumer preferences, from whole heads to fresh-cut salads and baby-leaf mixes. Lettuce is prized for its high water content, low calorie value, and nutritional benefits, offering essential vitamins, minerals, antioxidants, and dietary fiber [3,4,5]. Despite its widespread popularity, lettuce remains a highly perishable crop, susceptible to postharvest losses due to wilting, enzymatic discoloration, and tissue deterioration [6], which can be exacerbated by biotic and abiotic stresses encountered during cultivation and processing.

Lettuce cultivation faces growing difficulties due to alterations in weather conditions. Shifting temperatures and changing precipitation patterns have intensified abiotic stresses such as heat, drought, and salinity, threatening both crop yields and quality [7]. Prolonged periods of high temperature can disrupt the growth and development of lettuce, leading to premature bolting [8], reduced head formation, and poor texture, while low temperatures significantly slow down lettuce development [9]. Drought stress can restrict water availability, disrupting key physiological processes vital for optimal growth [10,11]. Plants under stress show a decrease in water potential and turgor pressure, stomatal closure, and reduced cell expansion [12], which accelerates leaf senescence. Premature leaf senescence caused by water stress triggers an earlier shift to the plant’s reproductive phase, marked by nutrient reallocation, degradation of leaf pigments, and a decline in chlorophyll content [13,14]. Salinity stress, often associated with irrigation in arid and semi-arid regions, poses an additional challenge. The accumulation of salts in the soil affects water uptake, leading to osmotic stress, ion toxicity, and nutrient imbalances in plants [10,15]. Elevated salinity inhibits plant and root growth, seed germination, and leaf water content, while elevating sodium and chloride ion concentrations and promoting lipid peroxidation [16,17,18,19,20]. Therefore, it is crucial to develop new methods and adaptive strategies to safeguard production and guarantee a stable food supply in the face of these environmental shifts.

Silicon (Si) plays a pivotal role in enhancing plant resilience under various abiotic stress conditions, including temperature extremes [21,22], salinity [23,24], and drought [25,26]. Si is not considered an essential nutrient for most plants but provides significant benefits under stress. In environments characterized by high salinity, Si has been shown to mitigate ionic toxicity and osmotic stress by enhancing ion balance and water retention in plant tissues [24]. Under drought conditions, Si improves water-use efficiency by enhancing root growth and modifying the transpiration rate, enabling plants to maintain physiological processes under limited water availability [26]. Similarly, Si contributes to thermotolerance by stabilizing cellular structures and protecting photosynthetic machinery from heat-induced damage [22].

Research has demonstrated that the accumulation of Si in plant tissues enhances cell wall strength and surface hydrophobicity, reducing water loss and protecting against damage caused by oxidative stress. Additionally, Si-mediated regulation of stress-responsive genes and antioxidant enzyme activity further underscores its role as a stress mitigator [26,27,28,29]. These mechanisms collectively enable plants to better withstand challenging environmental conditions, ultimately improving their growth, yield, and quality. In addition to root uptake, foliar application of Si-containing solutions provides an efficient alternative fertilization method that enhances silicon accumulation in plants [26].

The objective of this study was to explore the potential of foliar application of exogenous Si as a strategy to enhance lettuce crop productivity under stressful environmental conditions.

## 2. Results

### 2.1. Effect of SiO_2_ Application on Plant Phenotypic Traits

**Figure 1:**
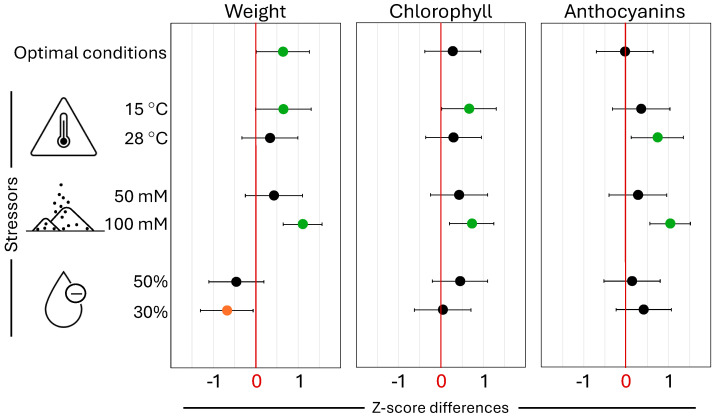
Effect of 3.66 mM foliar SiO_2_ application on lettuce fresh weight, chlorophyll, and anthocyanin content under seven environmental conditions. The study was conducted under optimal (20 °C), suboptimal (15 °C), and supraoptimal (28 °C) temperatures; moderate (50 mM NaCl) and severe (100 mM NaCl) salinity stress; and moderate (50% SWC) and severe (30% SWC) drought stress. Actual soil moisture content in drought treatments fluctuated from 30–40% to 50% and 20–25% to 30% SWC during watering intervals. Z-score differences, calculated across three cultivars and three experiments, represent the difference between SiO_2_-treated and control plants. Positive z-score differences indicate beneficial SiO_2_ effects, while negative z-scores indicate better control performance. Whiskers represent 95% confidence intervals. Green and orange circles denote statistically significant differences (*p* ≤ 0.05, Student’s *t*-test) above and below zero, respectively. Results for individual cultivars are in Supplemental Table S1. Each treatment (SiO_2_ and control) per environmental condition comprised 54 plants.

Silicon dioxide (3.66 mM SiO_2_) treatment enhanced plant biomass under all environmental conditions except drought, with significant increases (*p* < 0.05) observed under optimal (20 °C) (z-score difference = 0.643, *p* = 0.045) and suboptimal (15 °C) (z-score difference = 0.654, *p* = 0.048) temperatures, and under severe salinity (100 mM NaCl) (z-score difference = 1.107, *p* < 0.001) (Figure 1). However, 3.66 mM SiO_2_ treatment negatively impacted plant biomass under drought stress, with a non-significant decrease at 50% soil water content (SWC) (z-score difference = −0.460, *p* = 0.159) and a significant reduction at 30% SWC (z-score difference = −0.682, *p* = 0.033). Chlorophyll content increased across all tested conditions, with significant enhancement at both 15 °C (z-score difference = 0.665, *p* = 0.044) and severe salinity (100 mM NaCl) (z-score difference = 0.727, *p* = 0.008). Anthocyanin accumulation was elevated under all stress conditions, with significant increases observed at supraoptimal temperature (28 °C) (z-score difference = 0.737, *p* = 0.021) and under severe salinity (100 mM NaCl) (z-score difference = 1.040, *p* < 0.001). Analysis of plant biomass, chlorophyll content, and anthocyanin accumulation revealed that SiO_2_ treatment most consistently enhanced plant performance under severe salinity stress (100 mM NaCl), with significant positive effects across all measured parameters. This robust protective response to salinity stress suggests that optimizing SiO_2_ concentration could provide an effective strategy for mitigating salt-induced damage in plants. Significant SiO_2_ treatment × cultivar interaction was detected for chlorophyll content at 30% SWC (*p* = 0.011) and for anthocyanins content at 15 °C (*p* = 0.026) (Supplemental Table S1). Interactions close to significant level were also found for anthocyanins at 20 °C (*p* = 0.052) and plant fresh weight at 28 °C (*p* = 0.058).

### 2.2. Effect of Increasing SiO_2_ Concentrations to Alleviate Salinity Stress

**Figure 2:**
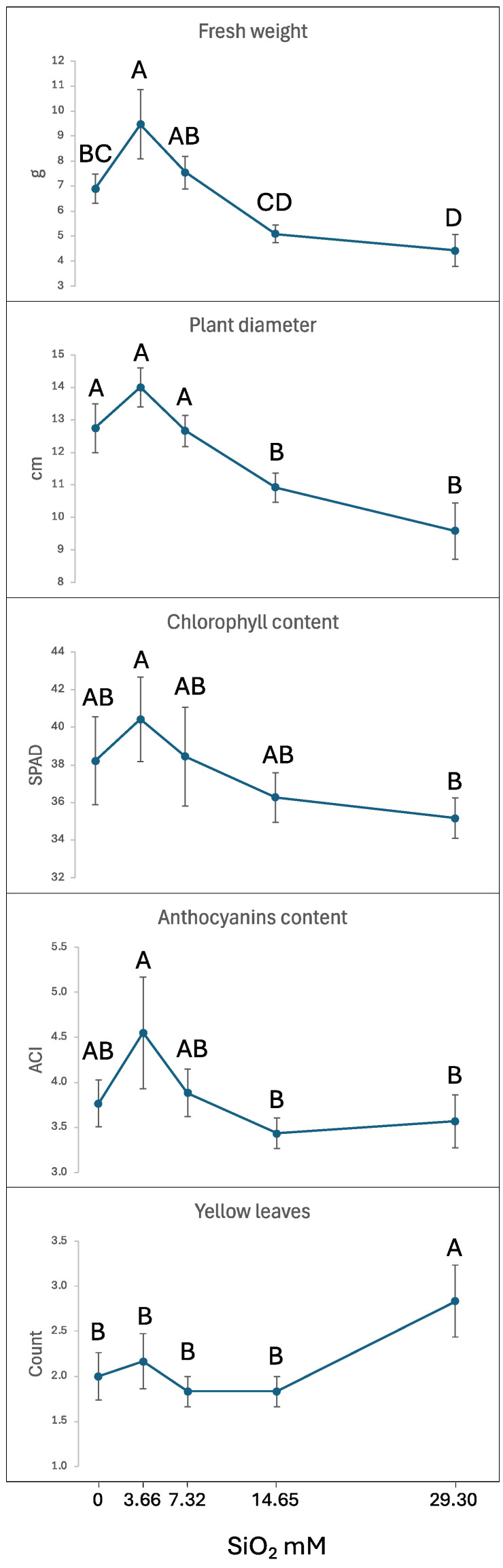
Effect of increasing SiO_2_ concentrations on lettuce performance parameters under severe salinity stress (100 mM NaCl). Silicon (or distilled water) was applied as a foliar spray at concentrations of 0, 3.66, 7.32, 14.65, and 29.30 mM. Plants were evaluated five weeks after planting. Each treatment comprised six plants. Values within each trait followed by different letters are significantly different (*p* ≤ 0.05) based on Tukey’s HSD test. Whiskers represent 95% confidence intervals.

**Figure 3:**
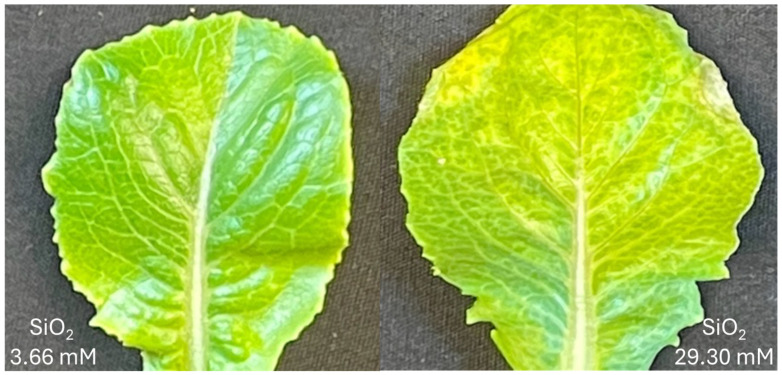
Comparison of lettuce leaves (cultivar Darkland) showing lowest and highest SiO_2_ treatment effects under salinity stress. (**Left**): leaf from plant sprayed with 3.66 mM SiO_2_; (**right**): leaf from plant sprayed with 29.30 mM SiO_2_. High concentrations of silicon under 100 mM NaCl stress caused substantial leaf chlorosis and early necrosis.

To identify the optimal SiO_2_ concentration for salt stress protection at 100 mM NaCl, we exposed plants to increasing doses of SiO_2_ (0, 3.66, 7.32, 14.65, and 29.30 mM). Phenotypic measurements across treatments showed ranges in fresh weight (4.4–9.5 g), plant diameter (9.6–14.0 cm), chlorophyll content (35.2–40.4 SPAD units), anthocyanin content (3.4–4.6 ACI units), and chlorotic leaf count (1.8–2.8). Four parameters—fresh weight, plant diameter, chlorophyll content, and anthocyanin levels—displayed similar dose-response patterns, peaking at 3.66 mM SiO_2_ before gradually declining at higher concentrations. In contrast, leaf chlorosis, an indicator of stress damage, reached its maximum at the highest SiO_2_ concentration (29.30 mM) (Figure 2 and Figure 3).

### 2.3. Effect of SiO_2_ Application on Mineral Composition

**Figure 4:**
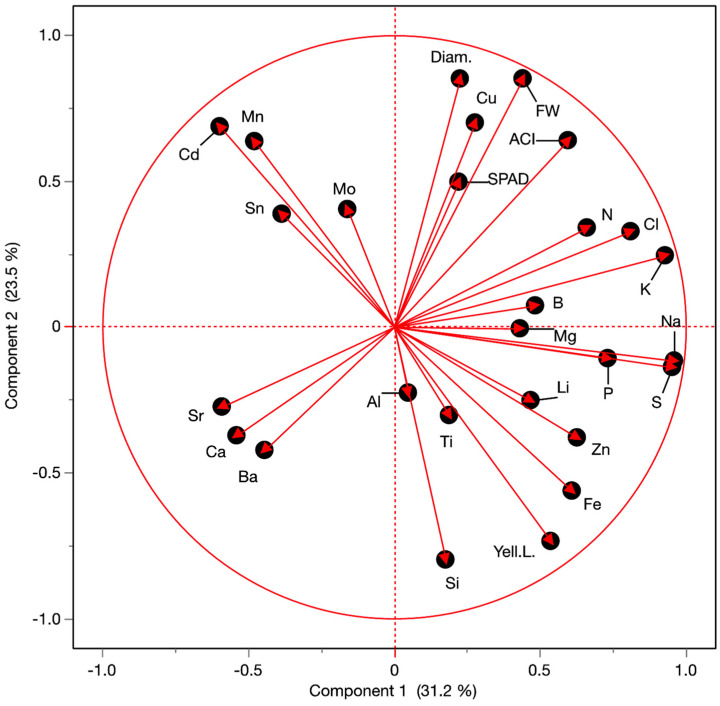
Principal component analysis of 22 mineral elements and five lettuce traits. Plants were grown under 100 mM NaCl salinity stress with foliar application of increasing SiO_2_ concentrations (0, 3.66, 7.32, 14.65, and 29.30 mM). Elements are indicated by their periodic table symbols. Lettuce traits are abbreviated as follows: FW (plant fresh weight), Diam. (plant diameter); SPAD (chlorophyll content), ACI (anthocyanin content); Yell.L. (number of chlorotic leaves).

We analyzed the mineral composition of 22 elements in plants exposed to severe salinity (100 mM NaCl) and varying SiO_2_ concentrations (Table 1). Principal component analysis (PCA) of mineral content and phenotypic traits revealed an association between leaf chlorosis and elevated silicon levels, with iron also showing a positive correlation (Figure 4). However, the Fe-chlorosis relationship appears spurious, as Fe concentrations showed minimal variation (33.3–42.0 mg/kg dry weight, DW) with no significant differences from control plants (Table 1). In contrast, tissue Si content increased dramatically with SiO_2_ treatment, reaching 103.6 mg/kg DW at 29.30 mM SiO_2_—a 77% increase compared to untreated controls (58.6 mg/kg DW). The PCA grouped fresh weight, plant diameter, anthocyanin content, and chlorophyll content together, consistent with the dose-response patterns observed in Figure 2. Notably, copper content clustered with these growth parameters (Figure 4) and reached its maximum (3.38 mg/kg DW) at 3.66 mM SiO_2_ (Table 1), the same concentration that optimized plant growth and physiological parameters.

**Figure 5:**
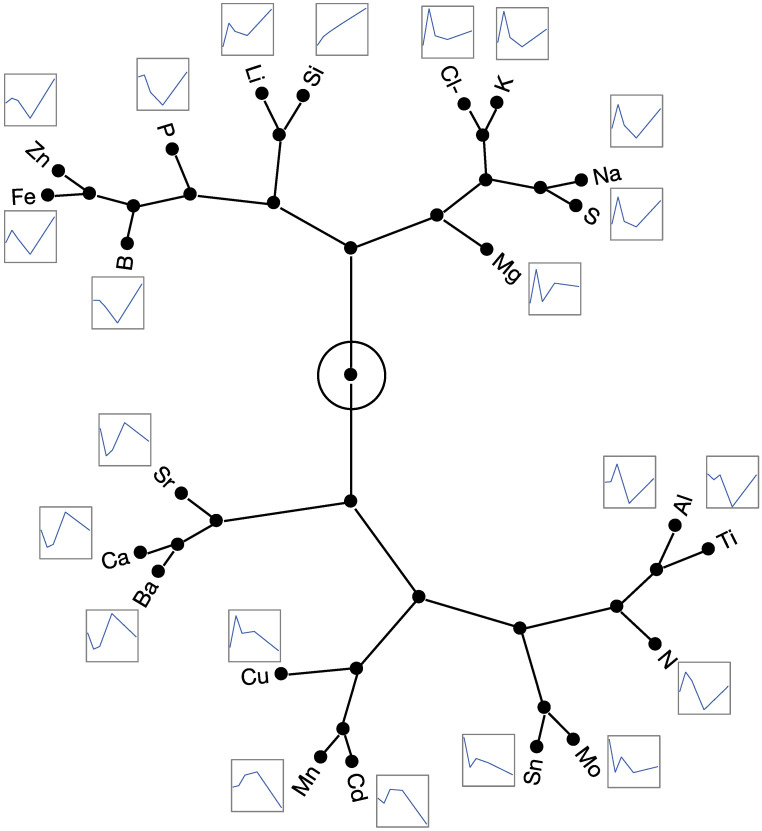
Hierarchical clustering of mineral elements composition based on their concentration-response profiles to foliarly applied SiO_2_. Samples were taken from plants grown under 100 mM NaCl salinity stress. The constellation plot represents the hierarchical clustering of element-specific response patterns to increasing SiO_2_ concentrations (0 to 29.30 mM), with concentration profiles displayed alongside each mineral element.

Hierarchical clustering of z-score transformed mineral composition data, which assessed the relative changes in elemental profiles independent of absolute concentrations across treatments, revealed distinct groupings of elements, suggesting similarities in their accumulation patterns in response to SiO_2_ foliar application (Figure 5). The strongest associations were observed among elements exhibiting the following profile patterns: Ca, Ba, and Sr (decrease, increase, and decrease again); Fe and Zn (increase, decrease, and increase again); Sn and Mo (sharp decrease from control); Cl^−^, K, Na, and S (peak at 3.66 mM SiO_2_); and Mn and Cd (sharp decline at the highest SiO_2_ treatment, 29.30 mM). These distinct profiles indicate that SiO_2_ foliar application influences the accumulation of certain mineral elements, possibly through shared uptake or transport mechanisms, or through related physiological responses within the plant.

## 3. Discussion

### 3.1. Silicon Uptake and Translocation Mechanisms

Plant species demonstrate remarkable variation in their silicon absorption and accumulation capacities, directly influencing their physiological responses to Si availability [30]. The classification system [31] categorizes plants into three distinct groups based on their Si accumulation patterns. Non-accumulators contain less than 0.5% Si dry mass and exhibit rejective uptake, where Si absorption occurs more slowly than water uptake. Intermediate accumulators accumulate 0.5–1% Si dry mass (or over 1% with a Si/Ca molar ratio below 1). Accumulators maintain over 1% Si dry mass with a Si/Ca molar ratio exceeding 1, indicating active uptake where Si absorption outpaces water absorption. This group predominantly includes grasses. While these distinct uptake modes (rejective, passive, active) are well-recognized, researchers continue investigating their precise underlying mechanisms [31].

#### 3.1.1. Active vs. Passive Transport Systems

Intermediate and accumulator species typically employ both passive and active Si uptake mechanisms [31]. Active uptake species, such as rice and wheat, utilize specialized influx and efflux transporters to efficiently move monosilicic acid into roots and transport it to the xylem. In contrast, passive uptake species—generally dicotyledons—primarily absorb Si along with water via the transpiration stream.

This difference is clearly illustrated by xylem Si concentrations where rice maintains 20- and 100-fold higher levels than cucumber and tomato, respectively. Rice achieves this through transporter-mediated loading, while cucumber and tomato rely on passive diffusion [32]. Interestingly, the balance between active and passive processes in plants like cucumber can shift depending on transpiration rates and Si demand [33].

#### 3.1.2. Transport and Distribution Mechanisms

When supplied via growth media, plants primarily absorb Si as monosilicic acid (H_4_SiO_4_) through their roots. This Si then travels to leaves via the xylem, largely driven by transpiration [34,35]. Transpiration plays a crucial role in Si movement throughout the plant, as water evaporation and symplastic water uptake efficiently concentrate Si, potentially leading to silica precipitation [36].

However, uncontrolled silica deposition can be detrimental to plant function, necessitating the evolution of regulated transport mechanisms [36]. While transpiration drives much of Si movement, especially for passive uptake, substantial evidence supports active uptake mechanisms involving specific Si transporters [37]. These active processes can achieve tissue Si concentrations exceeding those possible through purely passive transport, enabling Si accumulation even in plant parts with low transpiration rates [38].

This suggests active control over Si distribution that can decouple silicon transport from bulk water movement in some species [39]. Consequently, silicification in grasses involves multiple, cell-type-specific mechanisms: passive cell wall silicification, controlled cell wall silicification, and specialized silica cell formation [36].

#### 3.1.3. Benefits for Plant Performance

In Si accumulators, silicon application enhances plant growth and photosynthesis by improving structural integrity and reducing lodging, optimizing light capture, improving water use efficiency and preventing over-transpiration, preventing photosynthetic depression and chlorophyll degradation, and contributing to increased dry matter production [40].

Furthermore, Si significantly mitigates both abiotic stresses (drought, salinity, heavy metals) and biotic stresses (pathogens, pests) by forming physical barriers, improving water relations, boosting antioxidant defenses, and priming defense responses [34,35].

### 3.2. Silicon Dynamics in Lettuce: A Non-Accumulator Species

Lettuce is classified as a non-accumulator species. Typical Si concentrations in greenhouse-cultivated lettuce plants and roots range from 0.03–0.07% Si DW, while field-grown lettuce under non-stress conditions contains 0.03–0.13% Si DW in above-ground parts [41,42]. Notably, maximum concentrations of approximately 0.2% Si DW have been found in yellow spots associated with physiological disorders [43].

Despite field applications of calcium silicate (4000 kg ha^−1^) not significantly impacting Si content [44], non-accumulator species can still benefit from silicon, particularly under stress conditions [45]. Even small amounts of Si provide benefits through biochemical processes, signaling pathways, enhanced antioxidant activity, and improved nutrient uptake [46]. Examples include improved copper tolerance in snapdragon and enhanced salinity tolerance in tomato with Si supplementation [47,48]. Conversely, under non-stress conditions, only a minimal effect beyond increased Si concentration in shoots has been observed when Si supplements are provided to non-accumulator species [49].

#### 3.2.1. Foliar Application: An Alternative Approach

Our study supplied Si through foliar spray rather than root uptake. Foliar Si application offers a viable alternative, particularly for intermediate and non-accumulator crops like lettuce, by bypassing inefficient root uptake [31].

Foliar-applied silicon compounds, such as SiO_2_, are generally converted to bioavailable monosilicic acid before uptake occurs. This monosilicic acid is then absorbed through the leaf surface, predominantly via stomatal pores, with limited penetration through the cuticle for very small molecules [50]. Uptake efficiency depends on factors including solution pH, contact time, and the presence of penetration enhancers.

A meta-analysis of results from multiple studies demonstrated that Si significantly increased water movement, particularly stomatal conductance, in stressed plants (salinity and drought) but showed no consistent impact—and sometimes caused reduction—on water movement in unstressed plants, especially non-Poales species [51].

#### 3.2.2. Limited Mobility and Localized Effects

Once absorbed, silicon’s movement within the plant is generally limited due to restricted phloem mobility [35]. For example, in potatoes, foliar Si application resulted in the greatest Si concentration within leaves, whereas soil application increased Si concentration in leaves, stems, and roots [52].

This limited systemic mobility means a significant portion of foliar-applied Si remains within treated leaves, contributing to localized effects such as increased density of epidermal cells, cuticle thickening [53], and altered linkages in non-cellulosic polymers and lignin, which strengthens cell walls [54].

Foliar Si application has also been shown to increase chlorophyll content, leaf biomass, and enhance photosynthesis by increasing stomatal conductance [54]. Our study’s tissue Si content analysis confirmed effective absorption of foliar-applied SiO_2_ by lettuce leaves, demonstrating that lettuce leaves provide effective surfaces for Si uptake.

### 3.3. Effects of Silicon on Lettuce Cultivation and Physiology

Silicon application in lettuce cultivation across hydroponic and field systems shows potential benefits for growth, stress tolerance, and post-harvest quality through various methods including fertilizers, nutrient solution addition, foliar sprays, and seed priming. However, outcomes can vary with conditions and concentrations.

#### 3.3.1. Growth and Yield Enhancements

Si addition frequently improves lettuce growth in hydroponic systems. Rice husk silica increased fresh weight by 26% [55], while potassium silicate enhanced shoot biomass and chlorophyll content [56]. Generally, Si supplementation is considered beneficial for hydroponic lettuce production [57]. However, some hydroponic studies under normal, non-stress conditions report inconsistent yield benefits or even adverse effects at high Si concentrations [58,59,60].

Foliar sodium silicate application in field conditions improved vegetative growth and yield while increasing Si content in lettuce heads [61]. Si fertilizers boosted leaf fresh weight under severe water scarcity and leaf dry weight across various irrigation levels [62].

#### 3.3.2. Stress Mitigation Capabilities

Silicon enhances lettuce resilience against both abiotic and biotic environmental stresses. Under salinity stress, Si improved relative water content (RWC) and chlorophyll levels, though not always reaching unstressed control levels [63]. Under water deficit, Si application enhanced growth, protected photosynthetic performance [64], and improved yield by sustaining photosynthetic rate, stomatal conductance, and RWC, resulting in cooler canopies [25]. Si also reduced heavy metal (Cd/Pb) accumulation in shoots and roots in contaminated fields [65] and mitigated Cd toxicity during seed germination [66]. Contrary, it was reported that Si application can even exacerbate salinity effects [23,58].

Si improved resistance to downy mildew (*Bremia lactucae*) through seed priming [67] or hydroponic application [68], and reduced nematode (*Meloidogyne incognita*) populations while decreasing oxidative stress [56]. Conversely, no effect on Pythium root disease (*Pythium myriotylum*) severity was observed [59].

#### 3.3.3. Photosynthesis and Post-Harvest Quality

Si influences lettuce physiology beyond biomass accumulation. TiSiO_4_ nanoparticles in hydroponics enhanced chlorophyll levels and, at higher doses, stimulated photosystem II efficiency and electron transport rate [69]. Post-harvest benefits include extended shelf life through reduced water loss and increased leaf firmness during storage [55,70]. Si application also leads to beneficial compositional changes, such as lower nitrate accumulation and higher antioxidant activity [60].

### 3.4. Study Design and Rationale

The current study examined the performance of lettuce plants treated with 3.66 mM SiO_2_ under seven different environmental conditions. While three cultivars were tested, the study does not aim to evaluate the effects of Si treatment on each cultivar separately. Instead, it focuses on assessing the overall impact of Si treatment across all three cultivars. This strategy enables a generalized understanding of how Si influences plant performance at a broader level, accounting for potential cultivar differences while emphasizing the collective response to Si treatment. Pooling data from all cultivars allows for the identification of overarching trends, providing valuable insights into the potential of Si as a treatment for improving plant growth and stress resistance, irrespective of specific cultivar variations.

#### 3.4.1. Silicon-Mediated Response to Temperature

Foliar SiO_2_ application significantly (*p* < 0.05) enhanced plant weight under suboptimal (15 °C) and optimal (20 °C) temperature conditions (Figure 1). While a non-significant trend towards enhancement was noted at supraoptimal (28 °C). These findings align with previous studies demonstrating similar positive effects across various crop species [28,71]. Silicon’s protective mechanisms likely operate through multiple pathways, such as cell wall strengthening [72], heat shock protein accumulation [73], maintenance of ROS-antioxidant homeostasis [28], reduction in stomatal conductance [74], and stabilization of chlorophyll content [75]. The pigment-involved mechanism was particularly evident in our study, where silicon treatment significantly increased chlorophyll content at 15 °C and anthocyanin levels at 28 °C, suggesting temperature-specific protective responses. In previous studies, under greenhouse conditions, silicon treatment enhanced chlorophyll content, quantum efficiency of photosystem II, and levels of phenolic compounds and ascorbic acid [56].

#### 3.4.2. Silicon-Mediated Drought Stress Response

Despite well-documented benefits of Si treatment for drought stress resistance [5,29], our study found no positive impact when testing lettuce plants at 50% and 30% SWC under growth chamber conditions (Figure 1). In fact, under severe drought stress, foliar application of 3.66 mM SiO_2_ significantly decreased plant fresh weight compared to untreated controls. Although unexpected, similar ineffectiveness of silicon in alleviating drought stress has been reported in other crop species, including soybean [76], barley [77], tall fescue [78], and wheat [79]. Given silicon’s known effects on stomatal physiology [78], several mechanisms might explain these results: (1) excessive stomatal closure could have limited CO_2_ uptake, reducing photosynthesis and biomass accumulation; (2) under severe drought conditions, foliar-applied SiO_2_ may have adversely altered leaf osmotic potential; (3) silicon-induced reduction of ABA levels [77] might have intensified drought symptoms by impairing necessary stomatal closure; or (4) the formation of a physical barrier by foliar SiO_2_ application on leaf surfaces, interfering with light absorption, gas exchange, or cuticular water loss. While SiO_2_ film formation on surfaces upon drying is theoretically applicable to leaves, the complex nature of leaf surfaces introduces substantial variability, not explored in this or previous studies. It is important to note that the severity of response was genotype-specific. For instance, in our study, only one cultivar showed significant (z-score difference = −1.463, *p* = 0.003) decrease in weight with SiO_2_ treatment at 30% SWC, although all three exhibited similar trends (Supplemental Table S1). This aligns with previously reported genotype-specific effects [39], indicating the potential need for developing optimal SiO_2_ treatments for individual cultivars.

Comparing our results with previous studies reporting Si effect on drought stress is challenging due to differences in experimental design. These include variations in Si forms, application methods (foliar, soil, hydroponic), application frequency and concentration, growing substrates, drought stress levels, environmental conditions (temperature, humidity, photoperiod, light intensity), growing environments (field, greenhouse, growth chamber), fertilizers use, plant species, and other factors. Therefore, only general conclusions can be drawn. Moreover, as previously noted [79], a potential bias exists in interpreting Si effects on drought stress. Many studies attribute improved tolerance to Si even when observed benefits are not stress-specific and are also evident in control treatments [79].

#### 3.4.3. Silicon-Mediated Response to Salinity Stress

Foliar SiO_2_ treatment tended to show positive effects on plant weight, chlorophyll and anthocyanin content under both moderate (50 mM NaCl) and severe (100 mM NaCl) salinity treatments, though results were statistically significant only under severe salinity stress (Figure 1). These findings align with previous studies evaluating sodium silicate (Na_2_SiO_3_) for salinity stress mitigation in lettuce, where silicon supplementation reduced the negative impacts of salt stress and improved plant growth [16]. Similar beneficial effects have been documented across multiple crop species [24,29,71].

While the exact mechanisms remain under investigation, silicon appears to alleviate salt stress through multiple pathways, including reduced oxidative damage, decreased lipid peroxidation, enhanced photosynthetic capacity, and improved ion homeostasis [24]. Silicon increases antioxidative enzyme activity and enhances metrics associated with photosynthesis, water balance, and oxidative stress under salinity conditions [29]. Our results showing elevated levels of both chlorophyll and anthocyanins under severe salinity stress confirm the involvement of these pigments in silicon-mediated stress alleviation (Figure 1).

When testing increasing concentrations of SiO_2_ against 100 mM salinity stress, the 3.66 mM SiO_2_ treatment exhibited the highest values for fresh weight, plant diameter, chlorophyll and anthocyanin content compared to other tested concentrations (Figure 2), although these trends did not always reach statistical significance across all pairwise comparisons. Higher SiO_2_ concentrations applied foliarly under severe salinity stress gradually decreased all beneficial parameters from their peak at 3.66 mM SiO_2_ and increased chlorotic leaf occurrence at the highest concentration (29.30 mM SiO_2_). The mechanism behind this negative effect remains unclear—it could be directly related to elevated SiO_2_ concentrations, increased solution pH, osmotic stress, or the formation of a physical barrier on leaf surfaces interfering with photosynthesis. In parsley, another non-accumulator species, leaf shape distortion and tip necrosis were observed when Si was supplied via either a foliar spray or root drench, indicating a possible phytotoxicity [49]. The phytotoxic effect of silica nanoparticles on *Arabidopsis thaliana* is influenced by Si’s ability to increase pH, and significant toxicity was not observed upon adjusting the pH to levels optimal for plant growth [80]. In unrelated outdoor observations, distinct from the current growth chamber experiment, lettuce leaves treated with higher doses of SiO_2_ were thicker and appeared more rigid (Simko and Peng, unpublished data). These results parallel findings from hydroponic studies using sodium silicate, where optimal photo-synthetic rates occurred at 3.19 mM Na_2_SiO_3_, while higher concentrations inhibited both shoot and root growth in lettuce [57].

#### 3.4.4. Mineral Elements Composition

When analyzing plant mineral composition under severe salinity stress, copper (Cu) content clustered with growth parameters and pigment content (Figure 4), showing similar response patterns across treatments (Figure 2, Table 1). The highest Cu content (3.38 mg/kg DW) was observed at 3.66 mM SiO_2_, coinciding with peak values for fresh weight, plant diameter, chlorophyll, and anthocyanin content. This correlation aligns with Cu’s essential roles in chlorophyll synthesis, photosystem function, and anthocyanin production [81], as well as its involvement in cell expansion and division [82].

Mineral element content profiles under increased SiO_2_ treatments at severe salinity stress could be grouped based on their relative change (Figure 5). The most closely related grouping was observed for several elements, such as Sr, Ca, and Ba; Fe and Zn; Mn and Cd; and Cl^−^, K, Na, and S. The elements Sr, Ca, and Ba share similar uptake pathways [83], owing to their comparable ionic radii and charge, which allows them to be absorbed using similar transport mechanisms. Calcium channels and transporters facilitate the uptake of Sr and Ba [84], resulting in correlations between their concentrations. Iron (Fe) accumulation is often correlated with several other minerals such as zinc (Zn) due to shared uptake pathways, transport mechanisms, and physiological interactions [85]. Mn and Cd share uptake pathways through membrane proteins involved in metal ion transport, leading to a positive correlation [86]. Potassium (K) plays a key role in sulfate (SO_4_) uptake and its translocation within plants [87]. Under salinity stress, chloride (Cl^−^) and sodium (Na) accumulate in response to saline conditions, helping plants maintain osmotic balance [15]. Both Cl^−^ and K are highly mobile ions in plants and co-accumulate to maintain osmotic balance, particularly under salt stress [88].

Maintaining a high K/Na ratio in plant cells is essential for plant survival under NaCl stress [89]. In lettuce grown under non-saline conditions, reported K/Na ratios vary widely, ranging from approximately 4 [90] to almost 7 [42], exceeding 16 [91], and even surpassing 70 [92]. However, this ratio rapidly declines when plants are subjected to even mild salinity stress (10 mM NaCl; [92]). Minimum reported ratios under saline conditions include approximately 2.5 at 60 mM NaCl [92], less than 1 at 60 mM and 300 mM NaCl [90,91]. In studies applying 2 mM Si to plants under moderate and severe salinity, K/Na ratios were estimated to be approximately 1.5 and 1.0, respectively [58]. In our study, foliar application of 3.66 mM SiO_2_ resulted in the highest accumulation of both Na (16,185 mg/kg DW) and K (20,406 mg/kg DW) (Table 1), while maintaining the highest K/Na ratio (1.26). This corresponded with optimal plant growth parameters, including maximum fresh weight (9.47 g) and chlorophyll content (SPAD of 40.42), under severe salinity stress (Figure 2). K/Na ratios were lower at other SiO_2_ concentrations: 1.11 at 0 mM, 1.14 at 7.32 mM, and 1.12 at both 14.65 and 29.30 mM. The elevated K levels alongside increased Na and Cl^−^ suggest enhanced overall mineral uptake capacity at 3.66 mM SiO_2_ rather than impaired ion selectivity. This is further supported by the highest nitrogen content (1.62% DW) observed at this Si concentration, compared to control (1.37% DW) and higher Si treatments (1.14–1.51% DW). Indeed, maintaining a higher K/Na ratio despite increased absolute amounts of both ions is characteristic of successful stress adaptation [93]. This is further supported by the improved growth parameters and reduced chlorotic symptoms at this concentration. Our results suggest that SiO_2_ foliar treatment may enhance cellular ion compartmentalization rather than exclusion mechanisms, enabling plants to manage higher total ion content while minimizing cytoplasmic damage. However, the precise mechanism by which Si sprayed on the leaf surface is absorbed, transported within plants, and enhances plant performance under stress conditions remains unclear [94].

## 4. Materials and Methods

### 4.1. Plant Material and Experimental Setup

This study evaluated the effects of silicon (SiO_2_) treatment on three lettuce cultivars representing different horticultural types: Darkland (romaine), Salinas 88 (iceberg), and Tango (leaf type). Three seeds were sown per square plastic pot (10 × 10 cm at the top, narrowing at the base; depth: 8.89 cm). After germination, only one healthy seedling was maintained per pot. Four separate experiments were performed.

### 4.2. Growth Conditions

Lettuce plants were cultivated in a Conviron PGC-Flex growth chamber (Conviron, Pembina, ND, USA) under a 16-h light/8-h dark photoperiod at a light intensity of 1000 µmol/m^2^/s. Temperature varied according to the experimental treatment (see Section 2.3). Plants were fertilized every 7–10 days, beginning three weeks after planting, with Jack’s Classic 20-20-20 all-purpose water-soluble fertilizer with micronutrients (JR Peters, Allentown, PA, USA). A stock solution was prepared by dissolving 1.32 g of fertilizer in 1 L of distilled water. Approximately 1 mL of this solution was applied as a foliar spray to each plant.

Plants were grown in Premium Growers Mix potting soil (Sun Land Garden Products, Watsonville, CA, USA). Soil water content (SWC) was determined gravimetrically. Pots filled with soil were saturated with distilled water, weighed, and oven-dried at 105 °C to constant weight. SWC (%) was calculated as [(wet weight − dry weight)/dry weight] × 100. Optimal lettuce growth was defined at 75% SWC. Target drought levels were 50% and 30% SWC. However, actual SWC fluctuated during drought cycles, reaching minimums of approximately 30–40% and 20–25% before rewatering for moderate and severe drought treatments, respectively.

### 4.3. Silicon Treatment and Experimental Design

To assess the effect of Si on lettuce cultivars under various stress and control conditions, three independent experiments (1–3) were conducted. Three lettuce cultivars were subjected to seven growth conditions across these experiments. Each combination of experiment and growth condition, arranged in a randomized complete block design, included six plants per cultivar per treatment. A preliminary pilot study with SiO_2_ concentrations ranging from 1.83 to 29.30 mM SiO_2_ informed the selection of the treatment concentration. Plants were sprayed three times weekly (Monday, Wednesday, and Friday) with either a 3.66 mM SiO_2_ solution (Signature SST 28% Silica, Loveland Products, Greeley, CO, USA) or distilled water (control), with approximately 1 mL applied per plant. Spraying began one week post-emergence and continued until harvest. The seven growth conditions were:

- Optimal conditions (no stress): 20 °C; daily watering with distilled water to maintain 75% SWC.
- Suboptimal temperature: 15 °C; daily watering to maintain 75% SWC.
- Supraoptimal temperature: 28 °C; daily watering to maintain 75% SWC.
- Moderate salinity stress: 20 °C; watering with 50 mM NaCl solution.
- Severe salinity stress: 20 °C; watering with 100 mM NaCl solution.
- Moderate drought stress: 20 °C; watering every other day to maintain 50% SWC (with fluctuations as described above).
- Severe drought stress: 20 °C; watering every other day to maintain 30% SWC (with fluctuations as described above).

Plants grown under optimal temperature conditions (20 °C) were harvested after five weeks. Plants grown at 28 °C (due to faster growth) were harvested after four weeks, and those at 15 °C (due to slower growth) were harvested after seven weeks. At harvest, fresh weight, chlorophyll content (SPAD units), and total anthocyanin content (ACI units) were measured. Chlorophyll and anthocyanin content were measured using SPAD-502 (Konica Minolta, Tokyo, Japan) and ACM-2000 Plus (Opti-Sciences, Hudson, NH, USA) hand-held meters, respectively. Three measurements per leaf, taken approximately 2 cm from the tip (avoiding veins), were averaged before statistical analyses.

### 4.4. SiO_2_ Concentration and Salinity Stress

Experiment 4 evaluated the effects of increasing SiO_2_ concentrations (0, 3.66, 7.32, 14.65, and 29.30 mM) under severe salinity stress (100 mM NaCl) on cultivar Darkland. Approximately 1 mL of foliar sprays were applied thrice weekly on each of six plants per SiO_2_ concentration. At harvest, the following traits were measured: fresh weight, plant diameter, chlorophyll and anthocyanin content, number of chlorotic (yellow) leaves, and mineral composition (22 elements: Al, B, Ba, Ca, Cd, Cl^−^, Cu, Fe, K, Li, Mg, Mn, Mo, N, Na, P, S, Si, Sn, Sr, Ti, and Zn). Leaf surfaces were rinsed briefly with deionized water before analysis to remove any SiO_2_ deposits. The mineral composition was analyzed by Wallace Laboratories (El Segundo, CA, USA) using standard methods on oven-dried tissues (70 °C for approximately 24 h), as previously described [41]. All elements except nitrogen (expressed as % DW) are reported in mg/kg DW.

### 4.5. Statistical Analysis

For experiments 1–3, fresh weight, chlorophyll, and anthocyanin content were standardized within each experiment, cultivar, and environmental condition using z-scores: z = (X − μ)/σ, where X is the value to be standardized, μ is the mean and σ is the standard deviation of all values for that cultivar and environmental condition combination within the respective experiment. Student’s *t*-test was then used to determine significant differences between SiO_2_-treated and control plants under each environmental condition. The difference between standardized values (z-scores) of a given trait in SiO_2_ treated versus control plants was calculated. A positive z-score difference indicates that SiO_2_ application increases the trait value, while a negative z-score difference suggest better performance in untreated plants.

In experiment 4, ANOVA was employed to compare the effects of different SiO_2_ concentrations on fresh weight, plant diameter, chlorophyll and anthocyanin content, chlorotic leaf count, and mineral composition. Tukey’s HSD test was used for post hoc comparisons. Principal component analysis (PCA) and hierarchical clustering (HC) were applied to z-score transformed mineral composition data to explore elemental profile similarities. Statistical analyses were conducted using JMP Pro 18.1.0 (SAS Institute, Cary, NC, USA).

## 5. Conclusions

This study demonstrates the differential impact of foliar SiO_2_ application on lettuce under abiotic stress. A 3.66 mM SiO_2_ treatment significantly enhanced plant weight under optimal and low temperature, and also under high salinity (100 mM NaCl), where it boosted chlorophyll and anthocyanin and improved ion management (higher K/Na ratio). However, SiO_2_ negatively affected lettuce weight under severe drought, highlighting stress-specific responses. While these growth chamber findings underscore SiO_2_’s potential as a salinity stress mitigator in lettuce, field validation and mature crop assessment are crucial.

## Figures and Tables

**Table 1:** Elemental composition analysis of lettuce grown under severe salinity stress (100 mM NaCl) with increasing concentrations of foliarly applied SiO_2_ (0, 3.66, 7.32, 14.65, and 29.30 mM).

Element	Symbol	0 mM SiO_2_		3.66 mM SiO_2_		7.32 mM SiO_2_		14.65 mM SiO_2_		29.30 mM SiO_2_	
Aluminum	Al	32.2	-	32.4	-	39.3	-	24.0	-	33.6	-
Barium	Ba	2.54	-	2.42	-	2.44	-	2.68	-	2.51	-
Boron	B	13.2	-	13.2	-	13.0	-	12.5	-	13.7	-
Cadmium	Cd	0.204	AB	0.188	B	0.233	A	0.230	AB	0.120	C
Calcium	Ca	5639	-	5245	-	5313	-	6042	-	5627	-
Chloride	Cl^−^	31,054	C	36,989	A	32,610	BC	31,993	BC	33,393	B
Copper	Cu	2.73	C	3.38	A	3.02	B	3.06	B	2.66	C
Iron	Fe	36.0	AB	38.9	AB	36.9	AB	33.3	B	42.0	A
Lithium	Li	0.511	-	0.586	-	0.560	-	0.547	-	0.630	-
Magnesium	Mg	2036	B	2482	A	2057	B	2298	AB	2254	AB
Manganese	Mn	87.8	AB	88.4	AB	92.8	A	94.2	A	79.1	B
Molybdenum	Mo	0.337	-	0.188	-	0.257	-	0.187	-	0.214	-
Nitrogen	N	1.37	AB	1.62	A	1.51	AB	1.14	B	1.44	AB
Phosphorus	P	3215	AB	3240	A	2996	BC	2813	C	3284	A
Potassium	K	16,015	C	20,406	A	16,733	BC	15,423	C	17,864	B
Silicon	Si	58.6	D	69.3	CD	75.0	BC	84.8	B	103.6	A
Sodium	Na	14,464	B	16,185	A	14,687	B	13,781	C	15,885	A
Strontium	Sr	18.3	-	16.4	-	16.8	-	18.7	-	17.4	-
Sulfur	S	777	B	980	A	797	B	757	B	953	A
Tin	Sn	1.55	A	0.98	B	1.15	AB	1.07	AB	0.84	B
Titanium	Ti	0.607	-	0.574	-	0.602	-	0.419	-	0.602	-
Zinc	Zn	31.2	AB	32.0	AB	31.6	AB	28.6	B	35.3	A

The content of elements, except for nitrogen, is reported in mg/kg dry weight (DW). Nitrogen is expressed as % dry weight. Values within each element followed by different letters are significantly different (*p* ≤ 0.05) based on Tukey’s HSD test. Dash indicates element values showing no significant differences across SiO_2_ treatments. Each treatment comprised six plants.

## Data Availability

The original contributions presented in the study are included in the article/Supplementary Materials, further inquiries can be directed to the author.

## Supplementary Materials

The following supporting information can be downloaded at: https://www.mdpi.com/article/10.3390/plants14121845/s1, Table S1: Impact of 3.66 mM foliar SiO_2_ treatment on lettuce fresh weight, chlorophyll (SPAD), and anthocyanin (ACI) content across seven environmental conditions.

## References

[1] Simko I., Hayes R.J., Mou B., McCreight J.D., Smith S., Diers B., Specht J., Carver B. (2014). Lettuce and Spinach. Yield Gains in Major US Field Crops (CSSA Special Publications).

[2] Davis W.V., Weber C., Wakefield H., Wechsler S. (2025). Vegetables and Pulses Outlook: April 2025. VGS-375. Economic Research Service Situation and Outlook Report, United States Department of Agriculture. https://ers.usda.gov/sites/default/files/_laserfiche/outlooks/111478/VGS-375.pdf?v=51427.

[3] Kim M.J., Moon Y., Tou J.C., Mou B., Waterland N.L. (2016). Nutritional value, bioactive compounds and health benefits of lettuce (*Lactuca sativa* L.). J. Food Compos. Anal..

[4] Simko I. (2024). Spatio-temporal dynamics of lettuce metabolome: A framework for targeted nutritional quality improvement. Plants.

[5] Yang X., Gil M.I., Yang Q., Tomás-Barberán F.A. (2022). Bioactive compounds in lettuce: Highlighting the benefits to human health and impacts of preharvest and postharvest practices. Compr. Rev. Food Sci. Food Saf..

[6] Peng H., Simko I. (2023). Extending lettuce shelf life through integrated technologies. Curr. Opin. Biotechnol..

[7] Pathak T.B., Maskey M.L., Dahlberg J.A., Kearns F., Bali K.M., Zaccaria D. (2018). Climate change trends and impacts on California agriculture: A detailed review. Agronomy.

[8] Rosental L., Still D.W., You Y., Hayes R.J., Simko I. (2021). Mapping and identification of genetic loci affecting earliness of bolting and flowering in lettuce. Theor. Appl. Genet..

[9] Simko I., Hayes R.J., Furbank R.T. (2016). Non-destructive phenotyping of lettuce plants in early stages of development with optical sensors. Front. Plant Sci..

[10] Angon P.B., Tahjib-Ul-Arif M., Samin S.I., Habiba U., Hossain M.A., Brestic M. (2022). How do plants respond to combined drought and salinity stress?—A systematic review. Plants.

[11] Shin Y.K., Bhandari S.R., Jo J.S., Song J.W., Lee J.G. (2021). Effect of drought stress on chlorophyll fluorescence parameters, phytochemical contents, and antioxidant activities in lettuce seedlings. Horticulturae.

[12] Farooq M., Wahid A., Kobayashi N., Fujita D., Basra S.M.A. (2009). Plant drought stress: Effects, mechanisms and management. Agron. Sustain. Dev..

[13] Behmann J., Steinrücken J., Plümer L. (2014). Detection of early plant stress responses in hyperspectral images. ISPRS J. Photogramm. Remote Sens..

[14] Lim P.O., Kim H.J., Gil Nam H. (2007). Leaf senescence. Annu. Rev. Plant Biol..

[15] Munns R., Tester M. (2008). Mechanisms of salinity tolerance. Annu. Rev. Plant Biol..

[16] de Souza Lemos Neto H., de Almeida Guimarães M., Sampaio I.M.G., de Araújo Hendges A.R.A., de Oliveira A.B., Filho S.M. (2018). Silicon (Si) reduces the effects of salt stress on germination and initial growth of lettuce (*Lactuca sativa* L.). Aust. J. Crop Sci..

[17] Eraslan F., Inal A., Savasturk O., Gunes A. (2007). Changes in antioxidative system and membrane damage of lettuce in response to salinity and boron toxicity. Sci. Hortic..

[18] Mohammadi P., Khoshgoftarmanesh A.H. (2014). The effectiveness of synthetic zinc (Zn)-amino chelates in supplying Zn and alleviating salt-induced damages on hydroponically grown lettuce. Sci. Hortic..

[19] Pérez-López U., Miranda-Apodaca J., Muñoz-Rueda A., Mena-Petite A. (2013). Lettuce production and antioxidant capacity are differentially modified by salt stress and light intensity under ambient and elevated CO_2_. J. Plant Physiol..

[20] Xu C., Mou B. (2015). Evaluation of lettuce genotypes for salinity tolerance. HortScience.

[21] Shilpha J., Manivannan A., Soundararajan P., Jeong B.R., de Mello Prado R. (2023). Heat stress mitigation by silicon nutrition in plants: A comprehensive overview. Benefits of Silicon in the Nutrition of Plants.

[22] Thakral V., Bhat J.A., Kumar N., Myaka B., Sudhakaran S., Patil G., Sonah H., Shivaraj S., Deshmukh R. (2021). Role of silicon under contrasting biotic and abiotic stress conditions provides benefits for climate smart cropping. Environ. Exp. Bot..

[23] de Souza Lemos Neto H., Guimarães M., Mesquita R.O., Gomes Sampaio I., de Araújo Hendges A.R.A., de Oliveira A.B. (2018). Silicon potential as attenuator of salinity effects on growth and post-harvest quality of lettuce. J. Agric. Sci..

[24] Dhiman P., Rajora N., Bhardwaj S., Sudhakaran S.S., Kumar A., Raturi G., Chakraborty K., Gupta O.P., Devanna B., Tripathi D.K. (2021). Fascinating role of silicon to combat salinity stress in plants: An updated overview. Plant Physiol. Biochem..

[25] Villa e Vila V., Marques P.A.A., Gomes T.M., Nunes A.F., Montenegro V.G., Wenneck G.S., Franco L.B. (2024). Deficit irrigation with silicon application as strategy to increase yield, photosynthesis and water productivity in lettuce crops. Plants.

[26] Wang M., Wang R., Mur L.A.J., Ruan J., Shen Q., Guo S. (2021). Functions of silicon in plant drought stress responses. Hortic. Res..

[27] Mandlik R., Thakral V., Raturi G., Shinde S., Nikolić M., Tripathi D.K., Sonah H., Deshmukh R. (2020). Significance of silicon uptake, transport, and deposition in plants. J. Exp. Bot..

[28] Souri Z., Khanna K., Karimi N., Ahmad P. (2021). Silicon and plants: Current knowledge and future prospects. J. Plant Growth Regul..

[29] Thorne S.J., Hartley S.E., Maathuis F.J. (2020). Is silicon a panacea for alleviating drought and salt stress in crops?. Front. Plant Sci..

[30] Hodson M., White P.J., Mead A., Broadley M. (2005). Phylogenetic variation in the silicon composition of plants. Ann. Bot..

[31] Ma J.F., Miyake Y., Takahashi E., Datnoff L.E., Snyder G.H., Korndörfer G.H. (2001). Silicon as a beneficial element for crop plants. Silicon in Agriculture.

[32] Mitani N., Ma J.F. (2005). Uptake system of silicon in different plant species. J. Exp. Bot..

[33] Faisal S., Callis K., Slot M., Kitajima K. (2012). Transpiration-dependent passive silica accumulation in cucumber (*Cucumis sativus*) under varying soil silicon availability. Botany.

[34] Epstein E. (1999). Silicon. Annu. Rev. Plant Biol..

[35] Raven J.A. (1983). The transport and function of silicon in plants. Biol. Rev..

[36] Kumar S., Soukup M., Elbaum R. (2017). Silicification in grasses: Variation between different cell types. Front. Plant Sci..

[37] Ma J., Yamaji N. (2008). Functions and transport of silicon in plants. Cell. Mol. Life Sci..

[38] Yamaji N., Ma J.F. (2014). The node, a hub for mineral nutrient distribution in graminaceous plants. Trends Plant Sci..

[39] McLarnon E., McQueen-Mason S., Lenk I., Hartley S.E. (2017). Evidence for active uptake and deposition of Si-based defenses in tall fescue. Front. Plant Sci..

[40] Agarie S., Agata W., Kubota F., Kaufman P.B. (1992). Physiological roles of silicon in photosynthesis and dry matter production in rice plants: I. Effects of silicon and shading treatments. Jpn. J. Crop Sci..

[41] Cho E., Gurdon C., Zhao R., Peng H., Poulev A., Raskin I., Simko I. (2023). Phytochemical and agronomic characterization of high-flavonoid lettuce lines grown under field conditions. Plants.

[42] Simko I., Zhao R. (2023). Phenotypic characterization, plant growth and development, genome methylation, and mineral elements composition of neotetraploid lettuce (*Lactuca sativa* L.). Front. Plant Sci..

[43] Peng H., Zhao R., Smith R., Simko I. (2022). Phenotypic and genetic analyses of yellow spot malady in lettuce. Sci. Hortic..

[44] de Andrade F.A., Junior O.A., Perini L.J., de Jesus Andrade C.G.T., Miglioranza É. (2016). Yield, nutritional state and silicon accumulation in lettuce cultivars fertilized with calcium silicate. Agron. Sci. Biotechnol..

[45] Rastogi A., Yadav S., Hussain S., Kataria S., Hajihashemi S., Kumari P., Yang X., Brestic M. (2021). Does silicon really matter for the photosynthetic machinery in plants…?. Plant Physiol. Biochem..

[46] Irfan M., Maqsood M.A., Rehman Hu Mahboob W., Sarwar N., Hafeez O.B.A., Hussain S., Ercisli S., Akhtar M., Aziz T. (2023). Silicon nutrition in plants under water-deficit conditions: Overview and prospects. Water.

[47] Frantz J.M., Khandekar S., Leisner S. (2011). Silicon differentially influences copper toxicity response in silicon-accumulator and non-accumulator species. J. Am. Soc. Hortic. Sci..

[48] Hoffmann J., Berni R., Hausman J.-F., Guerriero G. (2020). A review on the beneficial role of silicon against salinity in non-accumulator crops: Tomato as a model. Biomolecules.

[49] Tebow J.B., Houston L.L., Dickson R.W. (2021). Silicon foliar spray and substrate drench effects on plant growth, morphology, and resistance to wilting with container-grown edible species. Horticulturae.

[50] Puppe D., Sommer M., Sparks D.L. (2018). Experiments, uptake mechanisms, and functioning of silicon foliar fertilization—A review focusing on maize, rice, and wheat. Advances in Agronomy.

[51] Cooke J., Carey J.C. (2023). Stress alters the role of silicon in controlling plant water movement. Funct. Ecol..

[52] Pilon C., Soratto R.P., Moreno L.A. (2013). Effects of soil and foliar application of soluble silicon on mineral nutrition, gas exchange, and growth of potato plants. Crop Sci..

[53] Jang S.-W., Sadiq N.B., Hamayun M., Jung J., Lee T., Yang J.-S., Lee B., Kim H.-Y. (2020). Silicon foliage spraying improves growth characteristics, morphological traits, and root quality of *Panax ginseng* C.A.Mey. Ind. Crops Prod..

[54] Hussain S., Shuxian L., Mumtaz M., Shafiq I., Iqbal N., Brestic M., Shoaib M., Sisi Q., Li W., Mei X. (2021). Foliar application of silicon improves stem strength under low light stress by regulating lignin biosynthesis genes in soybean (*Glycine max* (L.) Merr.). J. Hazard. Mater..

[55] Frasetya B., Subandi M., Sofiani I. (2021). (Eds) The Effect of Silica Source Concentration to Improve Growth of lactuca sativa L. on Floating Hydroponic System.

[56] de Souza Alonso T.A., da Silva D.L., de Mello Prado R., Soares P.L.M., Tenesaca L.F.L., Ferreira R.J. (2022). Silicon promotes the control of *Meloidogyne incognita* in lettuce by increasing ascorbic acid and phenolic compounds. J. Pest Sci..

[57] de Souza Lemos Neto H., de Almeida Guimaraes M., Sampaio I.M.G., da Silva Rabelo J., dos Santos Viana C., Mesquita R.O. (2020). Can silicon (Si) influence growth, physiology and postharvest quality of lettuce?. Aust. J. Crop Sci..

[58] de Souza Lemos Neto H., de Almeida Guimarães M., Mesquita R.O., Sousa Freitas W.E., de Oliveira A.B., da Silva Dias N., Gomes-Filho E. (2021). Silicon supplementation induces physiological and biochemical changes that assist lettuce salinity tolerance. Silicon.

[59] Helms K.M., Dickson R.W., Bertucci M.B., Rojas A.A., Gibson K.E. (2023). Metal micronutrient and silicon concentration effects on growth and susceptibility to pythium root rot forhydroponic lettuce (*Lactuca sativa*). Horticulturae.

[60] Tazekand F.M., Ghasemi K., Roosta H. (2022). Effect of silicon on the quantity and quality of Batavia lettuce in soilless culture conditions. J. Soil Plant Interact..

[61] Abdalla K.A., Youssef S.M.S., Ibrahim M.F., Salama Y.A., Metwally A.A. (2024). Impacts of cobalt, selenium and silicon biofortification on the growth, productivity and nutritional value of lettuce. Egypt. J. Hortic..

[62] Çelik Y. (2023). Effects of different irrigation levels and varying doses of silicon applications on yield and some physiological parameters in lettuce cultivation. Acta Sci. Pol. Hortorum Cultus.

[63] Alkahtani M., Hafez Y., Attia K., Al-Ateeq T., Ali M.A.M., Hasanuzzaman M., Abdelaal K. (2021). *Bacillus thuringiensis* and silicon modulate antioxidant metabolism and improve the physiological traits to confer salt tolerance in lettuce. Plants.

[64] Hidalgo-Santiago L., Navarro-León E., López-Moreno F.J., Arjó G., González L.M., Ruiz J.M., Blasco B. (2021). The application of the silicon-based biostimulant Codasil^®^ offset water deficit of lettuce plants. Sci. Hortic..

[65] Li S., Zhang S., Ding X., Liao X., Wang R. (2013). Spraying silicon and/or cerium sols favorably mediated enhancement of Cd/Pb tolerance in lettuce grown in combined Cd/Pb contaminated soil. Procedia Environ. Sci..

[66] Pereira A.S., Bortolin G.S., Dorneles A.O.S., Meneghello G.E., do Amarante L., Mauch C.R. (2021). Silicon seed priming attenuates cadmium toxicity in lettuce seedlings. Environ. Sci. Pollut. Res..

[67] de Cássia Alves R., dos Santos Zucco M.F., Oliveira K.R., Checchio M.V., Franco C.A., Körösi K., Gratão P.L. (2022). Seed priming with silicon improves plant resistance to downy mildew (*Bremia lactucae*) in lettuce seedlings by intensifying antioxidant defense systems. Silicon.

[68] Garibaldi A., Gilardi G., Cogliati E.E., Gullino M.L. (2012). Silicon and increased electrical conductivity reduce downy mildew of soilless grown lettuce. Eur. J. Plant Pathol..

[69] Mariz-Ponte N., Sario S., Mendes R.J., Correia C.V., Moutinho-Pereira J., Correia C.M., Santos C. (2020). Silicon titanium oxide nanoparticles can stimulate plant growth and the photosynthetic pigments on lettuce crop. Agriculture.

[70] Galati V.C., Magalhães Marques K., Ascari Morgado C.M., Corrêa Muniz A.C., Cecílio Filho A.B., Mattiuz B.-H. (2015). Silicon in the turgidity maintenance of American lettuce. Afr. J. Agric. Res..

[71] Rastogi A., Tripathi D.K., Yadav S., Chauhan D.K., Živčák M., Ghorbanpour M., El-Sheery N.I., Brestic M. (2019). Application of silicon nanoparticles in agriculture. 3 Biotech.

[72] Nazaralian S., Majd A., Irian S., Najafi F., Ghahremaninejad F., Landberg T., Greger M. (2017). Comparison of silicon nanoparticles and silicate treatments in fenugreek. Plant Physiol. Biochem..

[73] Khan A., Khan A.L., Imran M., Asaf S., Kim Y.-H., Bilal S., Numan M., Al-Harrasi A., Al-Rawahi A., Lee I.-J. (2020). Silicon-induced thermotolerance in *Solanum lycopersicum* L. via activation of antioxidant system, heat shock proteins, and endogenous phytohormones. BMC Plant Biol..

[74] Sonobe K., Hattori T., An P., Tsuji W., Eneji E., Tanaka K., Inanaga S. (2009). Diurnal variations in photosynthesis, stomatal conductance and leaf water relation in sorghum grown with or without silicon under water stress. J. Plant Nutr..

[75] Haghighi M., Pessarakli M. (2013). Influence of silicon and nano-silicon on salinity tolerance of cherry tomatoes (*Solanum lycopersicum* L.) at early growth stage. Sci. Hortic..

[76] Ruppenthal V., Zoz T., Steiner F., do Carmo L.M., Castagnara D.D. (2016). Silicon does not alleviate the adverse effects of drought stress in soybean plants. Semin. Ciências Agrárias.

[77] Maillard A., Ali N., Schwarzenberg A., Jamois F., Yvin J.-C., Hosseini S.A. (2018). Silicon transcriptionally regulates sulfur and ABA metabolism and delays leaf senescence in barley under combined sulfur deficiency and osmotic stress. Environ. Exp. Bot..

[78] Vandegeer R.K., Zhao C., Cibils-Stewart X., Wuhrer R., Hall C.R., Hartley S.E., Tissue D.T., Johnson S.N. (2021). Silicon deposition on guard cells increases stomatal sensitivity as mediated by K^+^ efflux and consequently reduces stomatal conductance. Physiol. Plant..

[79] Thorne S.J., Hartley S.E., Maathuis F.J. (2021). The effect of silicon on osmotic and drought stress tolerance in wheat landraces. Plants.

[80] Slomberg D.L., Schoenfisch M.H. (2012). Silica nanoparticle phytotoxicity to *Arabidopsis thaliana*. Environ. Sci. Technol..

[81] Yruela I. (2009). Copper in plants: Acquisition, transport and interactions. Funct. Plant Biol..

[82] Burkhead J.L., Gogolin Reynolds K.A., Abdel-Ghany S.E., Cohu C.M., Pilon M. (2009). Copper homeostasis. New Phytol..

[83] White P.J. (2001). The pathways of calcium movement to the xylem. J. Exp. Bot..

[84] White P.J., Bowen H.C., Demidchik V., Nichols C., Davies J.M. (2002). Genes for calcium-permeable channels in the plasma membrane of plant root cells. Biochim. Biophys. Acta (BBA)-Biomembr..

[85] Connorton J.M., Balk J., Rodríguez-Celma J. (2017). Iron homeostasis in plants–a brief overview. Metallomics.

[86] Thomine S., Vert G. (2013). Iron transport in plants: Better be safe than sorry. Curr. Opin. Plant Biol..

[87] Ródenas R., García-Legaz M.F., López-Gómez E., Martínez V., Rubio F., Ángeles Botella M. (2017). NO_3_^−^, PO_4_^3−^ and SO_4_^2−^ deprivation reduced LKT1-mediated low-affinity K^+^ uptake and SKOR-mediated K^+^ translocation in tomato and Arabidopsis plants. Physiol. Plant..

[88] White P.J., Broadley M.R. (2001). Chloride in soils and its uptake and movement within the plant: A review. Ann. Bot..

[89] Reddy I.N.B.L., Kim S.-M., Kim B.-K., Yoon I.-S., Kwon T.-R. (2017). Identification of rice accessions associated with K^+^/Na^+^ ratio and salt tolerance based on physiological and molecular responses. Rice Sci..

[90] Hniličková H., Hnilička F., Orsák M., Hejnák V. (2019). Effect of salt stress on growth, electrolyte leakage, Na^+^ and K^+^ content in selected plant species. Plant Soil Environ..

[91] Ondrasek G., Rengel Z., Maurović N., Kondres N., Filipović V., Savić R., Blagojević B., Tanaskovik V., Gergichevich C.M., Romić D. (2021). Growth and element uptake by salt-sensitive crops under combined NaCl and Cd stresses. Plants.

[92] Breś W., Kleiber T., Markiewicz B., Mieloszyk E., Mieloch M. (2022). The effect of NaCl stress on the response of lettuce (*Lactuca sativa* L.). Agronomy.

[93] Assaha D.V., Ueda A., Saneoka H., Al-Yahyai R., Yaish M.W. (2017). The role of Na^+^ and K^+^ transporters in salt stress adaptation in glycophytes. Front. Physiol..

[94] Pang Z., Peng H., Lin S., Liang Y. (2024). Theory and application of a Si-based defense barrier for plants: Implications for soil-plant-atmosphere system health. Crit. Rev. Environ. Sci. Technol..

