# Supplementary material for: Differential Impact of SiO2 Foliar Application on Lettuce Response to Temperature, Salinity, and Drought Stress
Source: Plants (Basel). 2025 Jun 16;14(12):1845. doi: 10.3390/plants14121845 (PMC12196634; doi:10.3390/plants14121845)
Supplement: Supplementary file 1 [file plants-14-01845-s001.zip › plants-3588736-supplementary.pdf]

Supplemental Table S1. Impact of 3.66 mM foliar SiO_2 treatment on lettuce fresh weight, chlorophyll (SPAD), and anthocyanin (ACI) content across seven environmental conditions.

Trait	Cultivar	20°C		15°C		28°C		50 mM NaCl		100 mM NaCl		50% SWC		30% SWC	
		Δ z	p-value	Δ z	p-value	Δ z	p-value	Δ z	p-value	Δ z	p-value	Δ z	p-value	Δ z	p-value
Weight	Darkland	0.511	0.402	0.593	0.371	-0.416	0.498	0.637	0.290	0.960	0.012	-0.475	0.437	-0.016	0.970
	Salinas	0.442	0.471	0.626	0.227	1.415	0.006	-0.510	0.294	1.022	0.007	0.098	0.874	-0.566	0.351
	Tango	0.976	0.090	0.744	0.112	0.007	0.991	1.163	0.036	1.340	0.003	-1.000	0.082	-1.463	0.003
	Combined	0.643	0.045	0.654	0.048	0.335	0.307	0.430	0.201	1.107	< 0.001	-0.460	0.159	-0.682	0.033
	Tr. × Cv. Inter.	-	0.770	-	0.945	-	0.058	-	0.077	-	0.657	-	0.738	-	0.171
SPAD	Darkland	0.572	0.345	1.365	0.013	0.242	0.695	0.326	0.597	0.634	0.114	0.695	0.246	0.712	0.234
	Salinas	0.931	0.109	-0.340	0.601	0.687	0.252	0.530	0.414	1.301	0.007	0.570	0.347	-1.341	0.011
	Tango	-0.662	0.271	0.953	0.099	-0.036	0.954	0.428	0.485	0.247	0.690	0.085	0.892	0.755	0.204
	Combined	0.280	0.394	0.665	0.044	0.298	0.365	0.429	0.202	0.727	0.008	0.450	0.167	0.042	0.899
	Tr. × Cv. Inter.	-	0.125	-	0.088	-	0.686	-	0.967	-	0.304	-	0.738	-	0.011
ACI	Darkland	1.095	0.052	1.222	0.035	0.747	0.209	0.619	0.305	0.948	0.013	1.151	0.039	1.093	0.052
	Salinas	-0.405	0.509	-0.882	0.137	0.658	0.247	-0.102	0.805	1.290	0.008	-0.275	0.656	0.056	0.928
	Tango	-0.781	0.188	0.729	0.222	0.805	0.173	0.305	0.621	0.883	0.130	-0.460	0.452	0.091	0.884
	Combined	-0.030	0.927	0.356	0.290	0.737	0.021	0.274	0.415	1.040	< 0.001	0.139	0.675	0.413	0.206
	Tr. × Cv. Inter.	-	0.052	-	0.026	-	0.983	-	0.670	-	0.724	-	0.100	-	0.358

Δ z: Z-score difference (SiO_2 treatment minus control). Positive values indicate SiO_2 benefit.

P-value: Student's t-test p-value.

Combined: Overall effect across cultivars.

Tr. × Cv. Inter.: Treatment × cultivar interaction p-value.

Green: SiO_2 treatment significantly higher ($p \leq 0.05$).

Orange: SiO_2 treatment significantly lower ($p \leq 0.05$).

Yellow: Significant ($p \leq 0.05$) treatment × cultivar interaction.
